# Accuracy of Interictal and Ictal Electric and Magnetic Source Imaging: A Systematic Review and Meta-Analysis

**DOI:** 10.3389/fneur.2019.01250

**Published:** 2019-12-03

**Authors:** Praveen Sharma, Margitta Seeck, Sándor Beniczky

**Affiliations:** ^1^Department of Clinical Neurophysiology, Danish Epilepsy Centre, Dianalund, Denmark; ^2^Department of Neurology, King George's Medical University, Lucknow, India; ^3^EEG & Epilepsy Unit, University Hospital of Geneva, Geneva, Switzerland; ^4^Department of Clinical Neurophysiology, Aarhus University Hospital and Department of Clinical Medicine, Aarhus University, Aarhus, Denmark

**Keywords:** EEG, epilepsy, ictal, interictal, MEG, presurgical evaluation, source imaging, source analysis

## Abstract

**Background:** Electric and magnetic source imaging methods (ESI, MSI) estimate the location in the brain of the sources generating the interictal epileptiform discharges (II-ESI, II-MSI) and the ictal activity (IC-ESI, IC-MSI). These methods provide potentially valuable clinical information in the presurgical evaluation of patients with drug-resistant focal epilepsy, evaluated for surgical therapy. In spite of the significant technical advances in this field, and the numerous papers published on clinical validation of these methods, ESI and MSI are still underutilized in most epilepsy centers performing a presurgical evaluation. Our goal was to review and summarize the published evidence on the diagnostic accuracy of interictal and ictal ESI and MSI in epilepsy surgery.

**Methods:** We searched the literature for papers on ESI and MSI that specified the diagnostic reference standard as the site of resection and the postoperative outcome (seizure-freedom). We extracted data from the selected studies, to calculate the diagnostic accuracy measures.

**Results:** Our search resulted in 797 studies; 48 studies fulfilled the selection criteria (25 ESI and 23 MSI studies), providing data from 1,152 operated patients (515 for II-ESI, 440 for II-MSI, 159 for IC-ESI, and 38 for IC-MSI). The sensitivity of source imaging methods was between 74 and 90% (highest for IC-ESI). The specificity of the source imaging methods was between 20 and 54% (highest for II-MSI). The overall accuracy was between 50 and 75% (highest for IC-ESI). Diagnostic Odds Ratio was between 0.8 (IC-MSI) and 4.02–7.9 (II-ESI < II-MSI < IC-ESI).

**Conclusions:** Our systematic review and meta-analysis provides evidence for the accuracy of source imaging in presurgical evaluation of patients with drug-resistant focal epilepsy. These methods have high sensitivity (up to 90%) and diagnostic odds ratio (up to 7.9), but the specificity is lower (up to 54%). ESI and MSI should be included in the multimodal presurgical evaluation.

## Introduction

### Rationale

In spite of the numerous published papers on the accuracy of electric source imaging (ESI) and magnetic source imaging (MSI) in localizing interictal epileptiform discharges and ictal activity, these methods have gained only partial acceptance in the presurgical evaluation of patients with drug-resistant focal epilepsy. A recently published survey by the E-PILEPSY consortium, comprising 25 European centers, showed that less than half of the centers used these methods for presurgical evaluation (1).

Interictal epileptiform discharges and ictal activity are typically recorded during long-term video EEG monitoring, which is part of the presurgical evaluation in almost all centers. However, signals are interpreted visually, without any post-processing or signal analysis. In the majority of centers, this is merely done by indicating the scalp region where the peak negativity of the discharges (phase reversal) is spotted. This can be misleading, since due to volume conduction, peak negativity can be recorded over a different lobe and even different side than the source. Interictal epileptiform discharges and ictal activity are essential components of the multimodal presurgical evaluation: they indicate the location of the irritative zone and the seizure-onset zone, respectively. Therefore, their accurate localization is extremely important for identifying the cortical area that needs to be surgically resected in order to render the patient seizure-free (2).

Source imaging methods estimate the location of the electric sources (ESI) and of the magnetic sources (MSI). Both methods can be applied for localizing interictal epileptiform discharges (II-ESI and II-MSI) and ictal activity (IC-ESI and IC-MSI). However, at present, MEG has the size of a scanner, and needs a shielded room together with special maintenance, requiring precisely scheduled recording times. Thus, restrictions in time (duration) and space (patient mobility) of the MEG recordings are inherent, so that IC-MSI is rarely performed.

Despite these limitations, during the last decades, these methods developed considerably. It is nowadays possible to record EEG and magnetoencephalographic (MEG) signals using high-density array, and individual head models are constructed using the patients' own MRI.

### Objectives

Our goal was to review the published literature on ESI and MSI in presurgical evaluation of patients with drug-resistant focal epilepsy and to infer its accuracy, from the published results.

We wanted to include a wide spectrum of methods and modalities: low density (LD) EEG recordings, with <64 electrodes, high density (HD) EEG recordings (64–256 electrodes), MEG recordings, analysis of interictal epileptiform discharges as well as of the ictal activity.

### Research Question

We have addressed the following question: What is the accuracy of electric and magnetic source imaging in the presurgical evaluation of patients with drug-resistant focal epilepsy?

## Methods

### Study Design

This is a systematic review and meta-analysis of the accuracy of electric and magnetic source imaging in presurgical evaluation.

### Participants, Interventions, Comparators

*Participants*: patients with drug-resistant focal epilepsy (3) who underwent presurgical evaluation for possible surgical treatment (resection).

*Interventions*: II-ESI (electric source imaging of interictal epileptiform discharges), IC-ESI (electric source imaging of ictal activity), II-MSI (magnetic source imaging of interictal epileptiform discharges) and IC-MSI (magnetic source imaging of ictal activity).

*Comparators*: The most widely accepted, clinically relevant gold standard (reference standard) for diagnostic methods in presurgical evaluation is the epileptogenic zone (EZ), inferred from the site of the resection and the postoperative outcome. Therefore, in this study we compared at sub-lobar level the location of the electric and magnetic sources with the resected area, and then the postoperative outcome (≥1 year after surgery).

### Systematic Review Protocol

Literature search was made for electric and magnetic source imaging studies in presurgical evaluation. We designed the review protocol, based on the PRISMA statement (Preferred reporting items for systematic reviews and meta-analyses) (4).

#### Search Strategy

We searched research studies published between January 1st 1991 and May 31st 2018. We restricted the search to human subjects that were published in English.

For ESI we used the following search string in PubMed and in EMBASE: (Epilepsy[Title/Abstract] AND Source imaging [Title/Abstract] AND Electric OR Electrical OR Electroencephalographic OR EEG [Title/Abstract]).

For MSI we used three different search strings in PubMed. String-1: (Epilepsy[Title] AND Magnetic [Title] OR MEG OR Magnetoencephalographic OR Electromagnetic OR[Title] AND Source Imaging[Title/Abstract]). String-2: (Ictal [Title] AND Magnetic Source Imaging [Title]). Sting-3: ((Magnetic source imaging[Title] OR Magnetoencephalography[Title]) AND Epilepsy[Title/Abstract] AND Interictal[Title/Abstract]).

Duplicate studies were eliminated.

#### Data Sources, Studies Selections, and Data Extraction

The studies were selected according to the following criteria: (1) Source Imaging compared with gold standard (as described in section Participants, Interventions, Comparators); (2) Studies with at least five subjects (up to four were included in case of ictal magnetic source imaging studies as there were very few studies); (3) follow up duration of minimum 1 year. First title and abstracts were screened, then the full text papers were screened and (for the selected papers) data were extracted, as detailed below.

The location of the epileptic focus indicated by the source imaging study was tested against gold standard to calculate accuracy parameters. If more than one source were found then the dominant one was used. All included patients underwent respective surgery. Resection of the source and seizure-freedom (Engel-1 outcome) ≥1 year after surgery was considered as evidence for correct localization of the epileptogenic zone, by the source imaging methods.

The definitions for accuracy parameters were used as follows: (a) Source imaging focus within resected area and Engel-1 outcome = True positive (TP); (b) Source imaging focus within resected area and outcome other than Engel-1 = False positive (FP); (c) Source imaging focus outside resected area and outcome Engel-1 = False negative (FN); (d) Source imaging focus outside resected area and outcome other than Engel-1 = True negative (TN). These data (TP, TN, FP, FN) were extracted from the selected studies.

In addition, for ESI studies we extracted information on the electrode array (low density vs. high density array).

### Data Analysis

Using the data extracted from the selected studies, we calculated the diagnostic accuracy measures, using the conventional formulae:

$$\begin{array}{l} Sensitivity=\frac{TP}{TP\;+\;FN} \end{array}$$

$$\begin{array}{l} Specificity=\frac{TN}{TN\;+\;FP} \end{array}$$

$$\begin{array}{l} Accuracy=\frac{TP\;+\;TN}{TP\;+\;TN\;+\;FP\;+\;FN} \end{array}$$

$$\begin{array}{l} Diagnostic\;Odds\;Ratio=\frac{TP*TN}{FP*FN} \end{array}$$

$$\begin{array}{l} Positive\;Likelihood\;Ratio=\frac{sensitivity}{1\;-\;specificity} \end{array}$$

$$\begin{array}{l} Negative\;Likelihood\;Ratio=\frac{1\;-\;sensitivity}{specificity} \end{array}$$

For all accuracy measures, we calculated 95% confidence intervals (CI). We compared the accuracy of the various source imaging methods (HD vs. LD recordings, interictal vs. ictal, ESI vs. MSI) using Chi square test, based on the numbers of TP, FP, TN, and FN. An open source software, OpenMeta[Analyst] was used to calculate accuracy parameters with statistical analysis (OpenMeta[Analyst]([Windows],[CEBM@BROWN],[USA],[2018]).

## Results

### Study Selection and Characteristics

**Figure 1:**
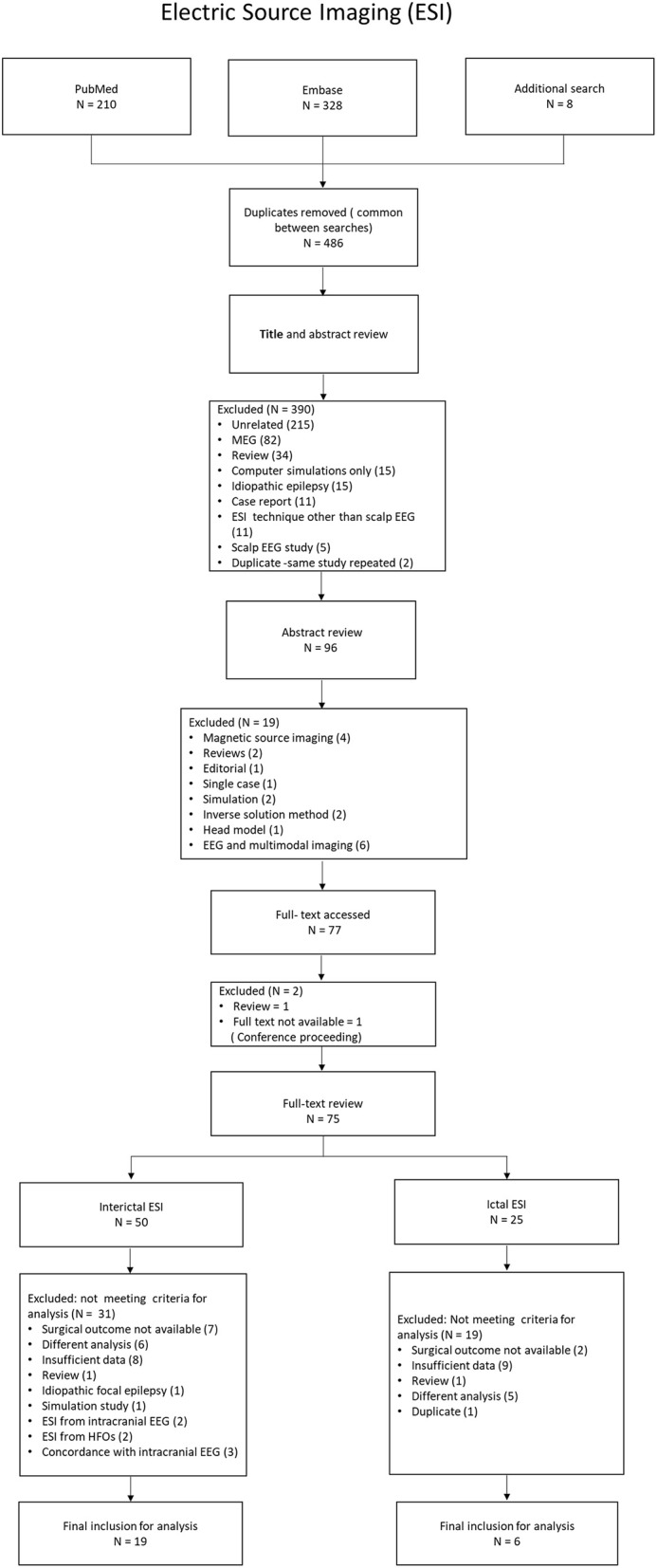
Flow diagram of the studies on ESI, retrieved for the review.

**Figure 2:**
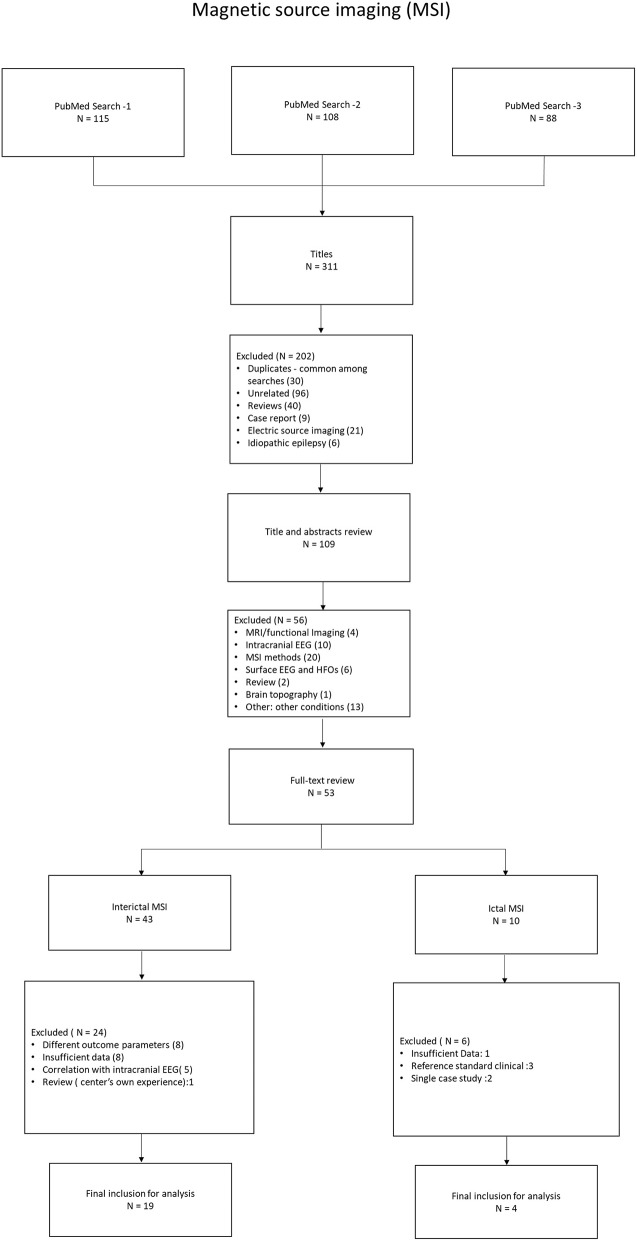
Flow diagram of the studies on MSI, retrieved for the review.

Figures 1, 2 show the flow diagram of the studies on ESI and MSI, retrieved for the review.

Our search strategy resulted in 486 ESI studies and 311 MSI studies, after removal of duplicates (Supplementary Material 1). After screening the titles and the abstracts, 77 ESI studies and 53 MSI studies were selected for full text review. Forty-eight fulfilled all selection criteria. Twenty-five of them addressed ESI: 19 studies on II-ESI (5–23) and six on IC-ESI (24–29). Twenty-three studies addressed MSI: 19 on II-MSI (30–48) and four on IC-MSI (49–52). Data were extracted from these studies for the meta-analysis. These were cross-sectional cohort studies (Supplementary Materials 2, 3).

From the studies selected for data extraction 15 ESI and eight MSI were prospective, including three IC-ESI and two IC-MSI studies, respectively. II-ESI studies included 11 studies with HD electrode array (64 or more electrodes, 64–256), four with LD electrode array (<64) and four with both. The studies with LD electrode array used 26 electrodes in one, 32 electrodes in other one and variable number of electrodes ranging from 19 to 29 and 27 to 32 in the rest. II-MSI studies mostly were with HD sensors except two with 37 sensor MEG and one study did not comment about the density of sensors.

There were three II-ESI and two II-MSI studies which included pediatric population of <18 years age. Other studies had mixed age group population with age ranging from 1 to 75 years from study to study.

There were very few studies on IC-MSI with fewer number of subjects fulfilling inclusion criteria. The sample size was very small, hence results should be interpreted in light of this bias. Study by Badier et al. (50) on IC-MSI used two methods of source analysis which resulted in two different results. These two methods were included as independent studies for the purpose of analysis. Both methods were correlated with epileptogenic zone mapped by intracranial EEG which in turn was used to calculate accuracy parameters. This introduces a methodical heterogeneity and reduces significance of results from this sub-group (IC-MSI).

### Synthesized Findings

The pooled patient population for assessment of the accuracy of source imaging included 1,152 patients totally (Table 1).

**Table 1:** Diagnostic outcome measures from pooled data (95% CIs in parenthesis).

	**II-ESI**	**IC-ESI**	**II-MSI**	**IC-MSI**
Number of patients	515	159	440	38
Sensitivity	81.1% (76.2–85.2%)	89.9% (81.8–94.6%)	77.4% (71.5–82.3%)	73.8% (48.4–89.4%)
Specificity	45.2% (36.0–54.7%)	46.9% (30.5–63.9%)	54.1% (46.2–61.9%)	20.5% (7.1–46.8%)
Accuracy	74.17% (70.39–77.95%)	74.84% (68.1−81.59%)	70.68% (66.43–74.94%)	50.00% (37.71–69.43%)
Diagnostic odds ratio	4.02 (2.31–6.98)	7.896 (3.117–20.004)	4.54 (2.81–7.32)	0.823 (0.16–4.229)
Positive likelihood ratio	1.31 (1.12–1.54)	1.47 (1.149–1.881)	1.42 (1.204–1.672)	0.98 (0.735–1.305)
Negative likelihood ratio	0.383 (0.263–0.557)	0.218 (0.169–0.281)	0.395 (0.282- 0.555)	1.04 (0.708–1.539)

Figures 3–14 show forest plots of the diagnostic outcome measures (sensitivity, specificity, diagnostic odds ratio, positive and negative likelihood rations) of the selected studies and the pooled data, for the source imaging methods: II-ESI, IC-ESI, II-MSI, IC-MSI. Table 1 summarizes the diagnostic outcome measures, determined from the pooled data. Table 2 summarizes comparisons of the outcome measures among the source imaging methods.

**Figure 3:**
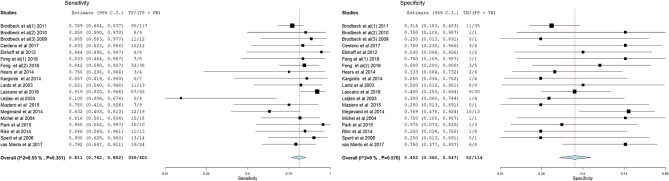
Forest plot showing sensitivity and specificity of the II-ESI studies (individual studies: size of the squares are proportional to weights used in meta-analysis; the summary measure: center line of the diamond; associated 95% confidence intervals: lateral tips of the squares and the diamond).

**Figure 4:**
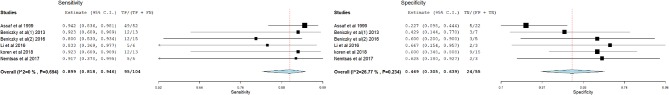
Forest plot showing sensitivity and specificity of the IC-ESI studies (individual studies: size of the squares are proportional to weights used in meta-analysis; the summary measure: center line of the diamond; associated 95% confidence intervals: lateral tips of the squares and the diamond).

**Figure 5:**
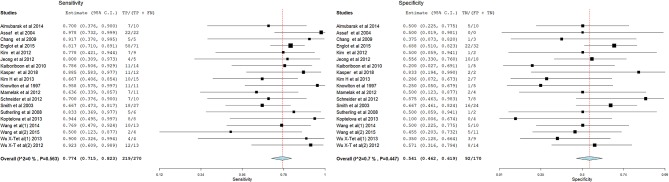
Forest plot showing sensitivity and specificity of the II-MSI studies (individual studies: size of the squares are proportional to weights used in meta-analysis; the summary measure: center line of the diamond; associated 95% confidence intervals: lateral tips of the squares and the diamond).

**Figure 6:**
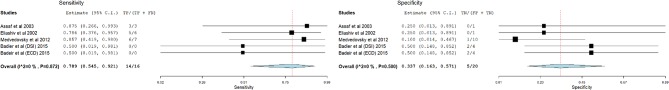
Forest plot showing sensitivity and specificity of the IC-MSI studies (individual studies: size of the squares are proportional to weights used in meta-analysis; the summary measure: center line of the diamond; associated 95% confidence intervals: lateral tips of the squares and the diamond).

**Figure 7:**
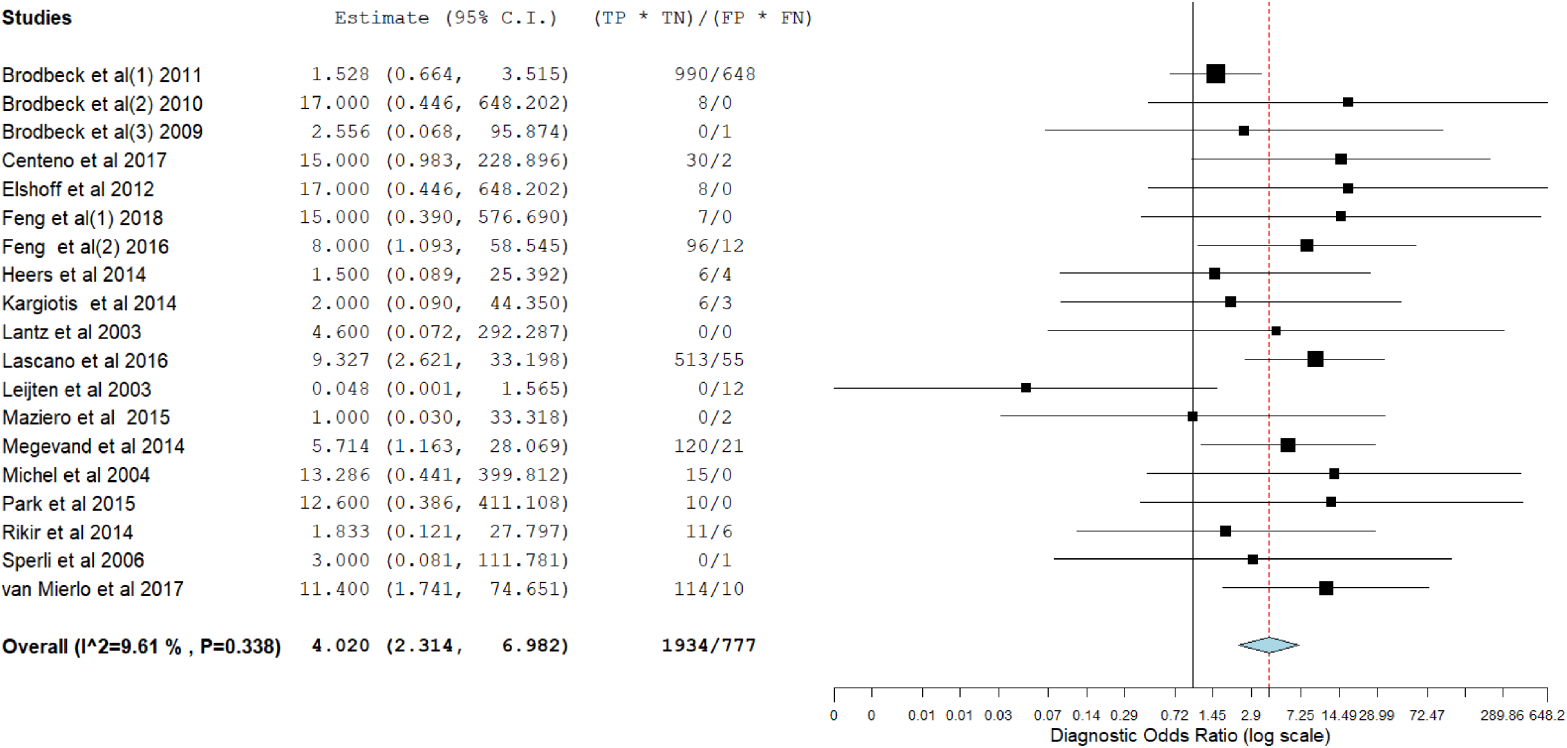
Forest plot showing diagnostic odds ratio of the II-ESI studies (individual studies: size of the squares are proportional to weights used in meta-analysis; the summary measure: center line of the diamond; associated 95% confidence intervals: lateral tips of the squares and the diamond).

**Figure 8:**
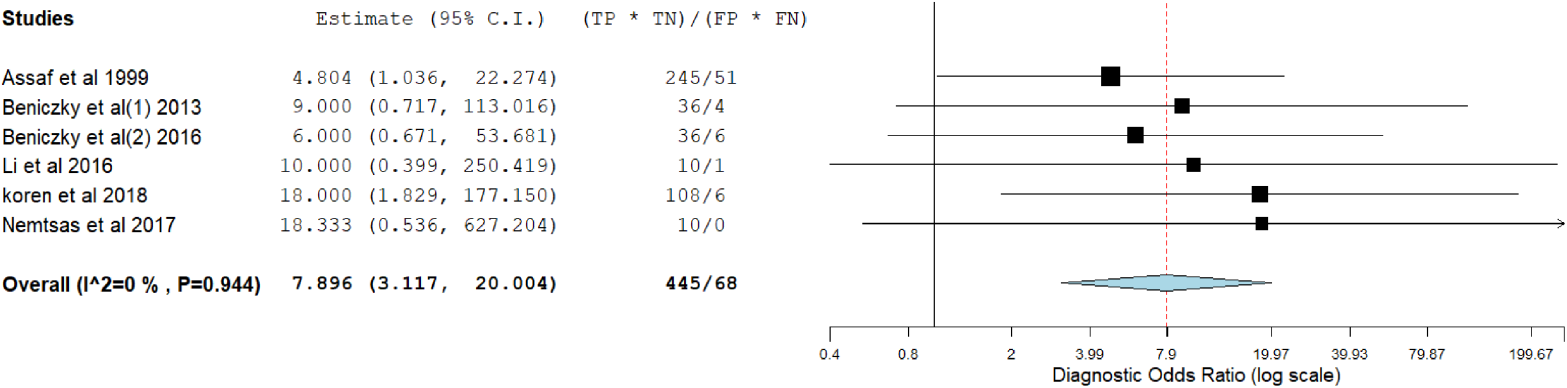
Forest plot showing diagnostic odds ratio of the IC-ESI studies (individual studies: size of the squares are proportional to weights used in meta-analysis; the summary measure: center line of the diamond; associated 95% confidence intervals: lateral tips of the squares and the diamond).

**Figure 9:**
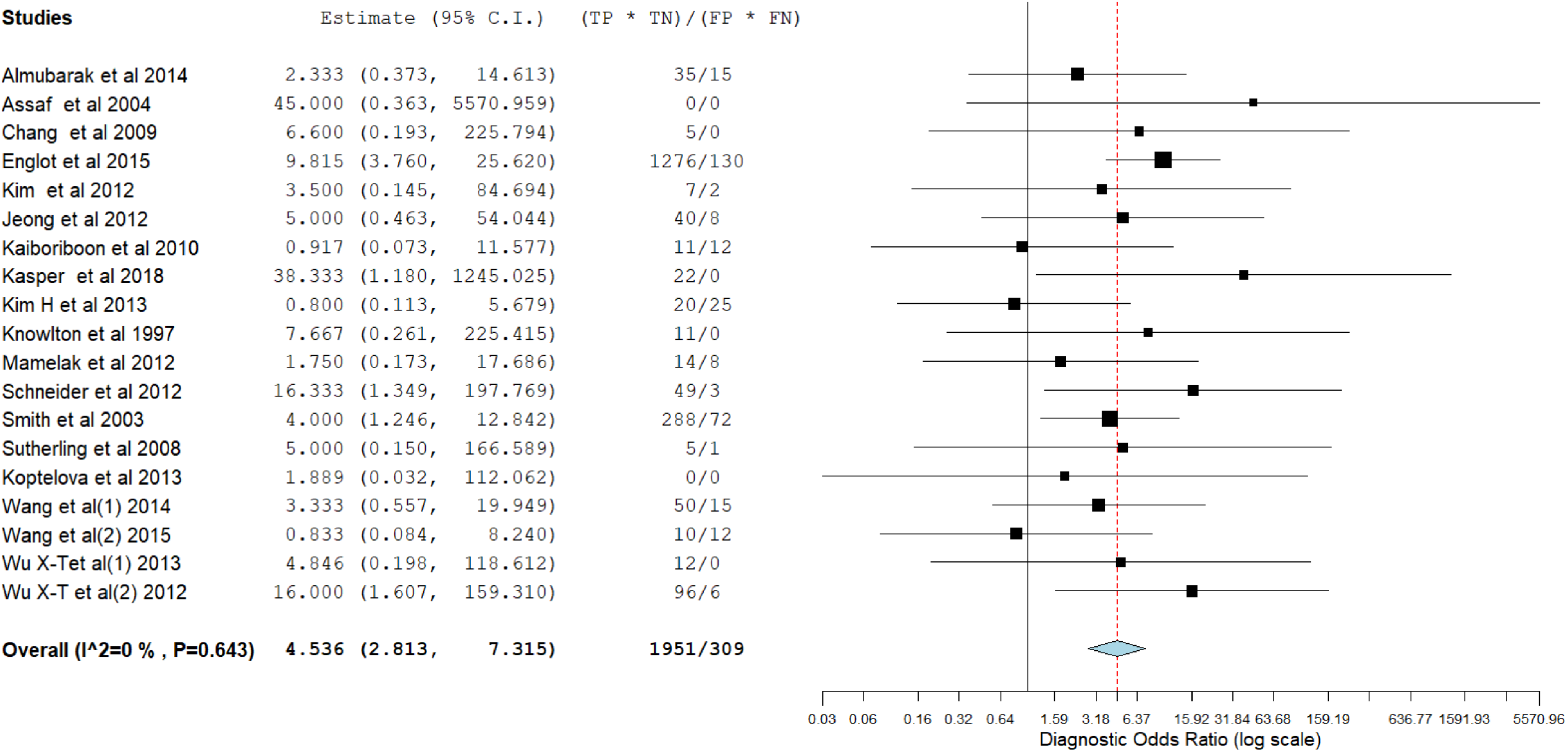
Forest plot showing diagnostic odds ratio of the II-MSI studies (individual studies: size of the squares are proportional to weights used in meta-analysis; the summary measure: center line of the diamond; associated 95% confidence intervals: lateral tips of the squares and the diamond).

**Figure 10:**
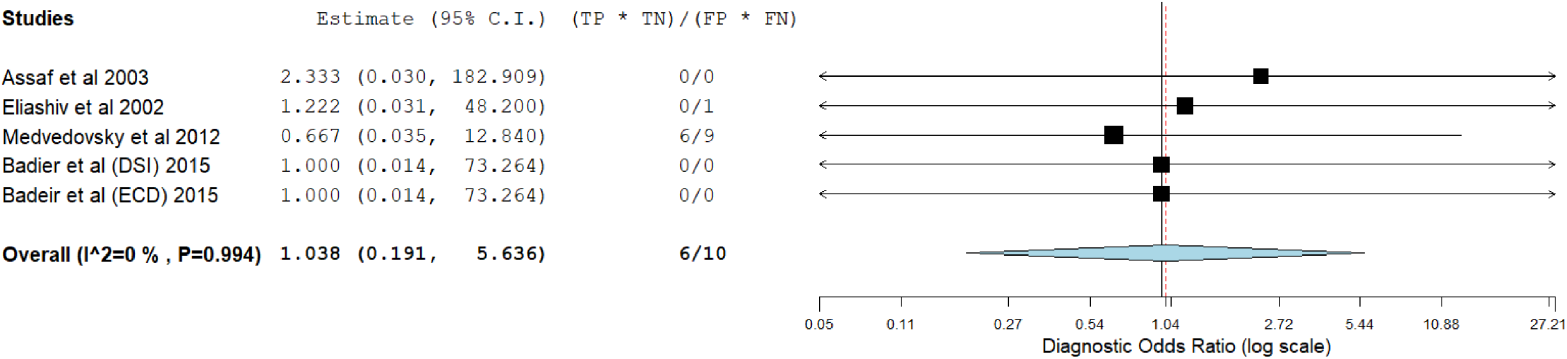
Forest plot showing diagnostic odds ratio of the IC-MSI studies (individual studies: size of the squares are proportional to weights used in meta-analysis; the summary measure: center line of the diamond; associated 95% confidence intervals: lateral tips of the squares and the diamond).

**Figure 11:**
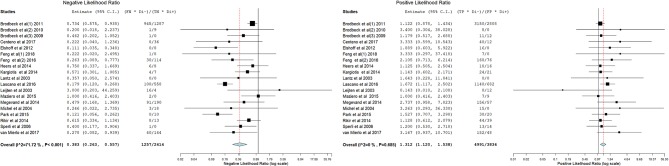
Forest plot showing negative and positive likelihood ratios of the II-ESI studies (individual studies: size of the squares are proportional to weights used in meta-analysis; the summary measure: center line of the diamond; associated 95% confidence intervals: lateral tips of the squares and the diamond).

**Figure 12:**
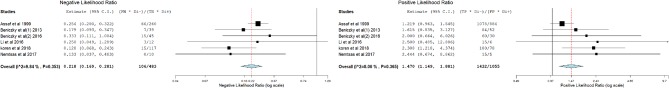
Forest plot showing negative and positive likelihood ratios of the IC-ESI studies (individual studies: size of the squares are proportional to weights used in meta-analysis; the summary measure: center line of the diamond; associated 95% confidence intervals: lateral tips of the squares and the diamond).

**Figure 13:**
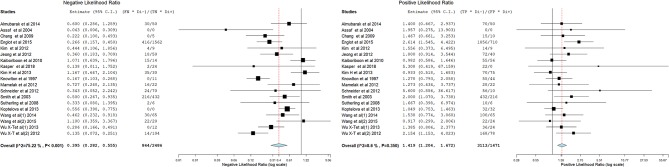
Forest plot showing negative and positive likelihood ratios of the II-MSI studies (individual studies: size of the squares are proportional to weights used in meta-analysis; the summary measure: center line of the diamond; associated 95% confidence intervals: lateral tips of the squares and the diamond).

**Figure 14:**
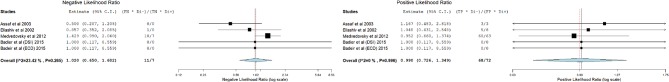
Forest plot showing negative and positive likelihood ratios of the IC-MSI studies (individual studies: size of the squares are proportional to weights used in meta-analysis; the summary measure: center line of the diamond; associated 95% confidence intervals: lateral tips of the squares and the diamond).

**Table 2:** Comparisons of the outcome measures among the source imaging methods.

	**Sensitivity (95%CI)**	***p***	**Specificity (95%CI)**	***p***	**Accuracy (95%CI)**	***p***
II-ESI	82.29% (78.56–86.03%)	**0.021**	45.61% (36.47–54.76%)	0.887	74.17% (70.39–77.95%)	0.865
IC-ESI	91.35% (85.94–96.05%)		43.64% (30.53–56.74%)		74.84% (68.10–81.95%)	
II-ESI	82.29% (78.56–86.03%)	0.697	45.61% (36.47–54.76%)	0.160	74.17% (70.39–77.95%)	0.22
II-MSI	81.11% (76.44–85.04%)		54.12% (46.63–45.61%)		70.68% (66.43–74.95%)	
IC-ESI	91.35% (85.94–96.05%)	0.056	43.64% (30.53–56.74%)	**0.023**	74.84% (68.10–81.95%)	**0.002**
IC-MSI	77.27% (59.76–94.17%)		12.50% (03.370–87.70%)		50.00% (34.10–65.95%)	
IC-MSI	77.27% (59.76–94.17%)	0.660	12.50% (03.370–87.70%)	**0.007**	50.00% (34.10–65.95%)	**0.0082**
II-MSI	81.11% (76.44–85.04%)		54.12% (46.63–45.61%)		70.68% (66.43–74.95%)	
II-ESI (HD)	84.21% (79.27–89.04%)	0.753	53.57% (40.51–46.63%)	0.964	77.74% (72.73–82.95%)	0.98
II-ESI (LD)	86.00% (76.38–95.09%)		52.94% (29.21–47.67%)		77.61% (67.63–87.95%)	
IC-ESI (HD)	85.00% (69.35–00.15%)	0.261	62.50% (28.95–37.05%)	0.245	78.57% (63.37–93.95%)	0.61
IC-ESI (LD)	92.86% (87.35–98.05%)		40.43% (26.40–59.46%)		74.05% (66.54–81.95%)	
II-ESI (HD)	84.21% (79.27–89.04%)	0.261	53.57% (40.51–46.63%)	0.962	77.74% (72.73–82.95%)	**0.0187**
II-MSI (HD)	80.00% (74.59–85.05%)		53.19% (44.96–46.43%)		69.23% (64.40–74.95%)	

*Significant differences are highlighted in bold*.

Sensitivity of the source imaging methods was between 73.8 and 89.9%, highest for IC-ESI and lowest for IC-MSI. Sensitivity was significantly higher for IC-ESI as compared with II-ESI (*p* = 0.02).

Specificity of the source imaging methods was between 20.5 and 45.1%, highest for II-MSI and lowest for IC-MSI. Specificity of IC-MSI was significantly lower compare with II-MSI (*p* = 0.007) and IC-ESI (*p* = 0.02).

The overall accuracy of the source imaging methods was between 50 and 74.84%, highest for IC-ESI and lowest for IC-MSI. Accuracy of the IC-MSI was significantly lower compared to the other methods (*p* < 0.002). There was no significant difference in accuracy between II-ESI, IC-ESI and II-MSI.

Diagnostic Odds Ratio was between 0.823 and 7.896, highest for IC-ESI and lowest for IC-MSI. The 95% CIs of all source imaging methods, except for IC-MSI, were >1.

Positive Likelihood Ratio was between 0.98 and 1.47, highest for IC-ESI and lowest for IC-MSI. Negative Likelihood Ratio was between 0.22 and 1.04, lowest for IC-ESI and highest for IC-MSI.

The sub-group analyses taking into account the spatial sampling, could not show significant difference in sensitivity, specificity or accuracy of II-ESI or IC-ESI, between HD and LD recordings (Table 2). However, the overall accuracy of II-ESI with HD recordings was significantly higher compared with II-MSI with HD recordings (*p* = 0.0187).

## Discussion

Based on a large number of operated patients (*n* = 1,152), the various EEG and MEG source imaging methods proved to have high accuracy in localizing the epileptic focus.

IC-MSI had the lowest performance, especially concerning its specificity. IC-ESI was done in a small number of patients (*n* = 38) due to its low feasibility (short recording time compared to EEG long term monitoring, and limited mobility of the patients with motor seizures in the MEG). These limitations might be overcome in future by the new generation of MEG equipment that allows room temperature measurements using optically-pumped magnetometers. Recently, EEG systems which allow EEG recordings of up to 256 electrodes for one or several days became commercially available. We hypothesize that there will be more studies on the yield of HD-EEG and HD-ESI with more widespread use of these systems, especially if electrode application and EEG analysis become easy to do.

Excluding the IC-MSI, the large pooled data showed high sensitivity of the source imaging methods, between 77 and 90%, highest for IC-ESI. However, their specificity was lower, between 45 and 54%, highest for II-MSI. The overall accuracy of the three methods was between 71 and 75% (highest for IC-ESI). Their Diagnostic Odds Ratio was between 4 and 7.9, highest for IC-ESI, demonstrating the diagnostic utility of these three source imaging methods.

The pooled data showed a significantly higher sensitivity of IC-ESI compared with II-ESI. From a technical point of view, IC-ESI is more challenging, due to the lower signal-to-noise ratio, difficulties in delimiting the ictal onset epoch, rapid propagation, electrodecremental response. However, our data suggest that these difficulties can be overcome, and that the gain from imaging the SOZ exceeds the errors potentially induced by the technical difficulties.

When restricting the analyses to the sub-groups of patients with HD EEG and HD MEG recordings, II-ESI had a higher diagnostic accuracy compared to II-MSI. The pooled data could not confirm higher accuracy of HD compared to LD ESI. It is important to emphasize the limitations in comparing accuracy of the source imaging methods, based on data pooled from different studies. There was a high heterogeneity in terms of study design and included patient populations among the studies, which could have biased the results of comparisons among the source imaging methods. Ideally, these methods should have been compared on the same patients (cross-over design) which was not the case here. Furthermore, most LD-recordings were obtained with >30 electrodes, which is not that low and might be sufficient for lesional epilepsy. Another important point is the underlying syndrome. Non-lesional extratemporal epilepsy may need HD-EEG/MEG to obtain correct localization, but this may be less relevant for tumoral temporal lobe epilepsy. Future studies may help to better stratify patients who need source localization with >64 electrodes or sensors

Our meta-analysis has several limitations. We did not limit our search strategy with any a priori assumption (for example, we did not exclude ictal studies or LD recordings). This rather inclusive strategy resulted in a high number of selected studies, but the drawback was the increased heterogeneity of study design, patient inclusion and source imaging methods, which potentially could have biased the results. Nevertheless, the large number of analyzed patients might have compensated for this, when inferring the main outcome results. The retrieval of published studies was limited to PubMed and EMBASE databases. We did not use Cochrane library. We restricted the studies that reported surgical standard as gold standard, thus excluding studies comparing source imaging with intracranial recordings. Strictly speaking, the best method of assessing the localization accuracy, *per se*, is comparing it with the intracranial recordings. However, the clinical relevance of this is questionable, since resecting the focus identified by intracranial recordings often does not lead to seizure-freedom. In addition, not all operated patients are implanted, which limits its use as a comparator for the whole group of operated patients. Furthermore, a systematic bias in all source imaging studies is the inclusion only of the operated patients. Although this is necessary for pragmatic reasons (need for gold standard), it potentially can lead to overestimation of the accuracy of the index test (source imaging). We grouped the methods according to the recorded modality (EEG vs. MEG) and the type of the analyzed EEG signals (interictal vs. ictal). This resulted in four categories, for which we calculated the accuracy measurements separately. Within each category, various types of inverse solutions were used for the analysis. However, recently published, large prospective and retrospective studies failed to prove significant difference I accuracy between the various inverse solutions (26, 53, 54).

Future studies need to address these limitations, with study design that overcome the issues listed above. There is a need for prospective, multi-center studies, with standardized electrode array and analysis pipeline (ideally as much automated as possible) and comparison of the various analysis methods within the same patients (cross-over design). Such a large, multi-center study has been recently initiated by European Reference Network (EpiCare), involving 20 epilepsy surgery centers.

Based on a large number of patients and studies, our results provide evidence for the accuracy of IC and II ESI and II MSI in localizing the epileptic focus. These methods should be included into the multimodal presurgical evaluation of patients with drug-resistant focal epilepsy.

## Data Availability Statement

The raw data supporting the conclusions of this manuscript will be made available by the authors, without undue reservation, to any qualified researcher.

## Author Contributions

PS, MS, and SB designed the study and contributed to editing the manuscript. PS and SB analyzed the data and drafted the manuscript.

### Conflict of Interest

MS has shares in Epilog, and received speaker fees by Philips. The remaining authors declare that the research was conducted in the absence of any commercial or financial relationships that could be construed as a potential conflict of interest.

## Supplementary Material

Supplementary Material 1 Results of the literature search.

Supplementary Material 2 Design of the included studies.

Supplementary Material 3 Characteristics of the included studies.
