# Supplementary material for: Accuracy of Interictal and Ictal Electric and Magnetic Source Imaging: A Systematic Review and Meta-Analysis
Source: Front Neurol. 2019 Dec 3;10:1250. doi: 10.3389/fneur.2019.01250 (PMC6901665; doi:10.3389/fneur.2019.01250)
Supplement: Supplementary Material 2 — Design of the included studies. [file Table_2.DOCX]

**Supplementary material 2: design of the included studies**

Prospective = P (n=20); retrospective = R (n=28)

| **S no.** | **Study category** | **Author** | **Year** | **Type (Prospective/Retrospective)** |
| --- | --- | --- | --- | --- |
| 1 | Inter-ictal ESI | Brodbeck et al | 2011 | P |
| 2 | Inter-ictal ESI | Brodbeck et al | 2010 | R |
| 3 | Inter-ictal ESI | Brodbeck et al | 2009 | R |
| 4 | Inter-ictal ESI | Cenetno et al | 2017 | P |
| 5 | Inter-ictal ESI | Elshof et al | 2012 | P |
| 6 | Inter-ictal ESI | Feng et al | 2018 | R |
| 7 | Inter-ictal ESI | Feng et al | 2016 | P |
| 8 | Inter-ictal ESI | Heers et al | 2014 | R |
| 9 | Inter-ictal ESI | Kargiotis et al | 2014 | R |
| 10 | Inter-ictal ESI | Lantz et al | 2003 | P |
| 11 | Inter-ictal ESI | Lascano et al | 2016 | P |
| 12 | Inter-ictal ESI | Leitzen et al | 2003 | P |
| 13 | Inter-ictal ESI | Mazerio et al | 2015 | R |
| 14 | Inter-ictal ESI | Megevand | 2014 | P |
| 15 | Inter-ictal ESI | Michel | 2004 | P |
| 16 | Inter-ictal ESI | Park et al | 2015 | P |
| 17 | Inter-ictal ESI | Rikir et al | 2014 | P |
| 18 | Inter-ictal ESI | Sperli et al | 2006 | R |
| 19 | Inter-ictal ESI | van Milero et al | 2017 | P |
| 20 | Ictal ESI | Assaf et al | 1999 | R |
| 21 | Ictal ESI | Benizcky et al(1) | 2013 | P |
| 22 | Ictal ESI | Benizcky et al (2) | 2016 | P |
| 23 | Ictal ESI | Li Chunsheng et al | 2016 | P |
| 24 | Ictal ESI | koren et al | 2018 | R |
| 25 | Ictal ESI | Nemtsas et al | 2017 | R |
| 26 | Inter-ictal MSI | Almubarak et al | 2014 | R |
| 27 | Inter-ictal MSI | Assaf et al | 2004 | P |
| 28 | Inter-ictal MSI | Chang et al | 2009 | R |
| 29 | Inter-ictal MSI | Englot et al | 2015 | R |
| 30 | Inter-ictal MSI | Kim H (1) et al | 2012 | R |
| 31 | Inter-ictal MSI | Jeong et al | 2012 | R |
| 32 | Inter-ictal MSI | Kaibariboon et al | 2010 | R |
| 33 | Inter-ictal MSI | Kasper et al | 2018 | R |
| 34 | Inter-ictal MSI | Kim H (2) et al | 2013 | R |
| 35 | Inter-ictal MSI | Knowlton et al | 1997 | P |
| 36 | Inter-ictal MSI | Mamelak et al | 2012 | P |
| 37 | Inter-ictal MSI | Schneider et al | 2012 | R |
| 38 | Inter-ictal MSI | Smith et al | 2003 | R |
| 39 | Inter-ictal MSI | Sutherling et al | 2008 | P |
| 40 | Inter-ictal MSI | Kaptelova et al | 2013 | P |
| 41 | Inter-ictal MSI | Wang et al (1) | 2014 | R |
| 42 | Inter-ictal MSI | Wang et al (2) | 2015 | R |
| 43 | Inter-ictal MSI | Wu XT et al (1) | 2012 | R |
| 44 | Inter-ictal MSI | Wu XT et al (2) | 2012 | R |
| 45 | Ictal MSI | Assaf et al | 2003 | R |
| 46 | Ictal MSI | Badier et al | 2015 | R |
| 47 | Ictal MSI | Eliashiv et al | 2002 | R |
| 48 | Ictal MSI | Medvedovsky et al | 2012 | R |
